# Breastfeeding and pain relief in full-term neonates during immunization injections: a clinical randomized trial

**DOI:** 10.1186/1471-2253-13-22

**Published:** 2013-09-13

**Authors:** Maryam Modarres, Azam Jazayeri, Parvin Rahnama, Ali Montazeri

**Affiliations:** 1 PhD student of Medical Education, Lecturer of Midwifery Education, Midwifery, Nursing & Midwifery Care Research Center, Tehran University of Medical Sciences (TUMS), Tehran, Iran; 2 Faculty of Nursing and Midwifery, Tehran University of Medical Sciences, Tehran, Iran; 3 Department of Midwifery, Shahed University, Tehran, Iran; 4 Mental Health Research Group, Health Metrics Research Center, Iranian Institute for Health Sciences Research, ACECR, Tehran, Iran

## Abstract

**Background:**

The aim of this study was to examine the effect of breastfeeding on pain relief in full-term neonates during injection of hepatitis B vaccine.

**Methods:**

This was a randomized clinical trial. A sample of full-term neonates was randomly allocated into two groups: the experimental group and the control group. Neonates in the experimental group were breastfed two minutes before, during, and after the hepatitis B immunization and the control group were held in mothers’ arms but not fed. Pain was assessed using the Douleur Aiguë du Nouveau-né (DAN) scale measuring facial expressions, limb movements and vocal expressions. The assessments were carried out after hepatitis B immunization.

**Results:**

One hundred thirty healthy full-term neonates were studied (65 in the experimental group and 65 in the control group). Gestational age, birth weight, Apgar score and gender did not differ between the two groups. The mean total pain score as measured by the DAN scale was 3.52 (SD = 1.37) for the experimental group and it was 6.78 (SD = 1.69) for the controls indicating a significant lower pain score for the experimental group (P<0.001). Also, there were significant differences for the three measures of DAN scale that are facial expressions, limb movements and vocal expression, between the two study groups (P<0.001).

**Conclusion:**

The findings confirm that breastfeeding reduces pain and is effective way for pain relief during hepatitis B vaccine injection.

**Trial registration:**

IRCT201104166206N1

## Background

Healthy neonates usually experience pain during the first week of life due to several medical procedures such as intramuscular injections and heal lancing [1]. Immunization injections are the most common source of pain in childhood [2]. Untreated pain early in life may cause harmful effects on the developing central nervous system [3] and also, it might exaggerate affective and behavioral responses during subsequent painful events [4,5]. The American Academy of Pediatrics recommended that:

‘*Every health care facility caring for neonates should implement an effective pain-prevention program, which includes strategies for routinely assessing pain, minimizing the number of painful procedures performed, effectively using pharmacologic and nonpharmacologic therapies for the prevention of pain associated with routine minor procedures, and eliminating pain associated with surgery and other major procedures’*[6,7].

It was suggested that pain management could be based on a “3-P” approach involving pharmacologic, physical and psychological strategies [8]. Thus, studies of pain relief in neonates still are a topic of interest in pediatrics. As such breastfeeding was found to be a safe and an effective method of pain relief in newborn babies [9-13].

Several reasons were suggested to explain why breastfeeding during painful procedures might reduce pain in neonates. These could be summarized as: maternal odor [14], antinociceptive effect [15] skin-to-skin contact [16], the sweet tasting of milk and the act of sucking [8].

The pain reduction methods that are used in painful procedures, including the use of oral sucrose, and pacifiers have been demonstrated [17,18]. However, these methods may interfere with a correct beginning of breastfeeding [19]. Therefore it is important to assess the analgesic effect of breastfeeding as a non-pharmacological and useful approach.

In the present study, we report the results from a randomized clinical trial that assessed the efficacy of breastfeeding for pain relief during injection of hepatitis B vaccination in term neonates.

## Methods

### Trial design

This was a randomized, controlled trial. The study was carried out in Mirza Kochak Khan Hospital, Tehran, Iran. The painful procedure used for this study was the hepatitis B vaccination.

### Participants

A sample of breastfeeding neonates was entered into the trial. Criteria for inclusion were: full- term neonate; had Apgar score of 7 and higher at five minutes after birth; delivered by spontaneous and vaginal delivery; were exclusively breastfed; postnatal age not more than 24 hours. We excluded infants with any evidence of congenital abnormalities, medical complications, or drug exposure.

### Study setting

This study was conducted in Mirza Kochak Khan Hospital, Tehran, Iran.

### Intervention

The study did not start until the infant was observed to be sucking at the breast. Neonates in the experimental group were breastfed during two minutes before, during, and after hepatitis B vaccination. At the end of the second minute of breastfeeding, while the infants were still sucking, an experienced nurse performed the immunization injections.

A dose of the vaccine (0.5 ml) was drawn into an Auto-disable (AD) syringe under the aseptic technique and then administered intramuscularly to the anterior thigh at a 90 angle to the skin with a 23-G, 1-inch needle. The Hepatitis B vaccine was manufactured by the Pasteur Institute of Iran and it preserved with Thimerosal. In order to minimize variability one experienced nurse, performed immunizations. For the controls the same procedure was applied while they were held in mothers’ arms but not fed. None of the infants had been breastfed for at least 30 minutes before the study procedures commenced. A trained research assistant using an electronic timer was responsible for timing of breast feeding or holding. To standardize the procedure a pilot trial with five neonates was performed.

### Outcome measure

The Douleur Aiguë du Nouveau-né (DAN) scale was used to assess pain. The scale was developed by Carbajal et al. in order to measure acute pain in newborn infants [10,20]. Scores on the scale range from 0 (no pain) to 10 (maximum pain) and measures three items: facial expressions, limb movements, and vocal expression (Table 1). We asked permission from Ricardo Carbajal to use the DAN in this study. Since several studies reported different timing for the assessment of pain [9-11], we performed a pilot study and found that behavioural changes were the most important measurements for assessing pain. Also we found that the best time for assessing pain would be 45 seconds from needle injection. Scoring was performed in real time. There was an observer (outcome assessor) to complete the outcome measure.

**Table 1:** **The Douleur Aigüe du Nouveau-né (DAN) scale**[10,20]

**Pain estimation**	**Score**
**Facial expressions**	
Calm	0
Snivels and alternates gentle eye opening and closing	1
Intensity of eye squeeze, brow bulge, nasolabial furrow:	
- Mild, intermittent with return to calm*	2
- Moderate†	3
- Very pronounced, continuous‡	4
**Limb movements**	
Calm or gentle movements	0
Intensity of pedalling, toes spread, legs tensed and pulled up, agitation of arms, withdrawal reaction:	
- Mild, intermittent with return to calm*	1
- Moderate†	2
- Very pronounced, continuous‡	3
**Vocal expression**	
No complaints	0
Moans briefly (for intubated child, looks anxious or uneasy)	1
Intermittent crying (for intubated child, expression of intermittent crying)	2
Long lasting crying, continuous howl (for intubated child, expression of continuous crying)	3

* Present during <1/3 of observation periods.

† Present during 1/3 to 2/3 of observation periods.

‡ Present during >2/3 of observation periods.

### Sample size

Based on a previous study [21], a minimum sample size of 60 infants per group (at least) was estimated. A study with such a sample size would have a power of 90% at a 0.05 significance level. However, we recruited 65 infants for each group, giving a total sample of 130 neonates.

### Randomization

**Figure 1:**
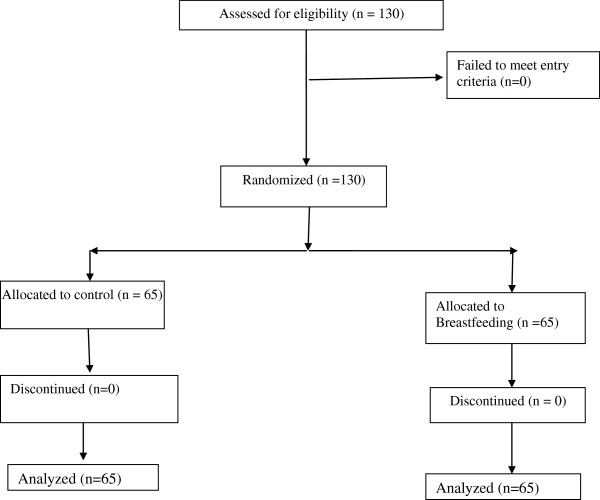
The trial flowchart.

Neonates were randomly assigned to the study groups. A system of sealed envelopes was used for assignment of the eligible neonates (Figure 1).

### Allocation concealment

The randomization code was available only to a research fellow who was not connected to the study. The code was disclosed to the researchers when the statistical analysis was completed.

### Blinding

The mothers and nurses were not blind to the group assignments. However, outcome assessor (observer) did not know the purpose and hypothesis of the study and the main investigator was blind to when the statistical analysis had been completed.

### Analysis

The SPSS version 16.0 was used to analyze the data. Descriptive analyses were carried out to explore the data. The chi square test used to compare categorical variables. The t test was used to compare pain scores between two groups. A significance level of alpha 0.05 or lower was adopted for all main analysis.

### Ethics

Approval for the study was obtained from the Office for Protection of Research Subjects in Tehran University of Medical Sciences. We obtained written informed consent from mother of each neonate included in the study.

## Results

### Neonate characteristics

In all 130 neonates were studied (65 in the experimental group and 65 in the control group). There were no significant differences between the two groups concerning perinatal characteristics including Apgar scores (P = 0. 20), gestational age (P = 0. 08), neonates’ weight (P = 0. 08), and gender (P = 0.86) (Table 2). The mean time since birth when the neonates actually received their injection was 12.0 (SD=4.03) hours.

**Table 2:** Perinatal characteristics of the study samples

	**Control group (n = 65)**	**Experimental group (n = 65)**	
	**Mean (SD)**	**Mean (SD)**	**P**
**Gestational age (weeks)**	39.4 (1.2)	39.1 (1.3)	0.08
**Birth weight (1000 gr.)**	3.59 (0.40)	3.55 (0.38)	0.50
**Apgar score**	8.8 (1.2)	7.0 (1.3)	0.20
**Boys/girls (numbers)**	30/35	29/36	0.80

### Effect of the intervention

There was significant difference in mean of facial expressions of neonates between the control and experimental groups (2.58 (SD=0.72), 1.39 (SD=0.65) respectively). (P<0.001, Table 3).

**Table 3:** Pain evaluation with the DAN scale (0–10) according to the facial expressions, limb movements and vocal expression

	**Control group (n = 65)**	**Experimental group (n = 65)**	
	**Mean (SD)**	**Mean (SD)**	P*
**Facial expressions**			
	2.58 (0.72)	1.39 (0.65)	<0.001
**Limb movements**			
	1.92 (0.69)	0.83 (0.51)	<0.001
**Vocal expression**			
	2.28 (0.57)	1.31 (0.68)	<0.001
**Total DAN Score**			
	6.78 (1.69)	3.52 (1.37)	<0.001

* Derived from t-test.

The results also showed that there was significant difference between the two groups in mean of limb movements 1.92 (SD=0.69) and experimental groups 0.83 (SD=0.51). (P<0.001, Table 3).

The results of this study showed that there were significant differences in mean of Vocal expression between control 2.28 (SD=0.57) and experimental groups 1.31 (SD=0.68). (P<0.001, Table 3). In addition as shown in Table 3 the difference of the DAN total score between two groups was significant.

Finally to have a better insight the DAN scores for each subscales that are facial expression, limb movements and vocal expressions are presented in Table 4.

**Table 4:** Pain evaluation with the DAN (0–10) in details (facial expression, limb movements and vocal expressions)

		**Control group (n = 65)**		**Experimental group (n = 65)**	
**Facial expressions**	**Scores**	**Frequency**	**%**	**Frequency**	**%**
	0	0	0	0	0
	1	3	4.6	4	6.2
	2	27	41.5	34	52.3
	3	29	44.6	25	38.5
	4	6	9.2	2	3.1
**Limb movements**					
	0	0	0	15	23.1
	1	18	27.7	36	70.8
	2	34	52.3	4	6.2
	3	13	20	0	0
**Vocal expression**					
	0	0	0	7	10.8
	1	4	6.2	32	49.2
	2	39	60	25	38.5
	3	22	33.8	1	1.5

## Discussion

Fortunately, women usually do breastfed their neonates in Iran. We thought this would be a good opportunity to assess whether we could confirm previous findings on the effectiveness of breastfeeding for pain relief during a hurting medical procedure such as immunization injections. The findings from this study revealed that breastfeeding was effective for pain relief in neonates during injection of hepatitis B vaccination. This study was a step forward among similar studies for two reasons. Firstly we assured if breastfeeding was real by observing sucking movement. Secondly, breastfeeding was begun two minutes before and continued during and after hepatitis B vaccination.

The finding from this study was very similar to the findings from other investigations [22]. For example, studies have shown that the breastfeeding effectively reduced response to pain during minor invasive procedure in term neonates [10] and significantly decreased crying in infants receiving immunization [12].

The role of facial expressions in measuring pain has been questioned by a recent study [23]. Therefore we reported the DAN subscales in addition to the total score to evaluate the role of each separately. However, the results showed that the three measures of DAN scale (facial expressions, limb movements, and vocal expression) were lower in the experimental group as compared to the controls.

It is argued that reactions to pain by nenonates are both physiological and psychological. Consistent with our study, a study revealed that the newborns who received breastfeeding demonstrated less physiologic and behavioral responses to pain in comparison with newborns held in their mother’s arms and not breastfed [1]. In addition studies have shown that breastfeeding, maternal holding, and skin to skin contact reduced crying in infants receiving an immunization injection [11].

The vaccine we used in this study was preserved with Thimerosal. In general it is argued that infant pain would be higher when using different physicochemical properties [24,25]. However, since we used the same vaccine for all the participants, thereby the possibility of bias could be ruled out.

As indicated it is interesting to know that most women (90%) do breastfed their neonates in Iran [26]. Despite such advantage unfortunately health centers do not use breastfeeding as a non-pharmacological intervention for pain relief for immunizations or other practices [27].

### Limitations

This study had some limitations. For instance selection of a single center and un-blinded nature of the study might influence the results. In addition we did not assess intra-rater reliability. Thus one should be cautious in generalizing the findings from this study. However, we were fortunate that all participants met the study criteria for the inclusion. One explanation for such compliance might be attributed to the pure chance. In addition one might question, for instance, how was that all women in this study had Apgar score of 7 or higher? The reason for this was due to the fact that all women in this study had vaginal delivery and we did not include any women who underwent caesarean section and therefore we did not consider this as failure or refusal.

## Conclusion

The findings from this study suggest that breastfeeding is effective in pain reduction in immunization procedure in neonates. It may be replace practices with procedures.

## Competing interests

The authors declared that they have no competing interests.

## Authors’ contributions

MM wrote the first draft. AJ was the main investigator, designed the study, collected the data, and performed analysis. PR helped the main investigator to analyze the data. AM contributed to the analysis, critically evaluated the paper, and provided the final draft. All authors read and approved the final version of the manuscript.

## Pre-publication history

The pre-publication history for this paper can be accessed here:

http://www.biomedcentral.com/1471-2253/13/22/prepub
